# Guidelines for the Management of Severe Traumatic Brain Injury: 2020 Update of the Decompressive Craniectomy Recommendations

**DOI:** 10.1093/neuros/nyaa278

**Published:** 2020-08-06

**Authors:** Gregory W J Hawryluk, Andres M Rubiano, Annette M Totten, Cindy O’Reilly, Jamie S Ullman, Susan L Bratton, Randall Chesnut, Odette A Harris, Niranjan Kissoon, Lori Shutter, Robert C Tasker, Monica S Vavilala, Jack Wilberger, David W Wright, Angela Lumba-Brown, Jamshid Ghajar

**Affiliations:** Section of Neurosurgery, GB1—Health Sciences Centre, University of Manitoba, Winnipeg, Manitoba, Canada; INUB-MEDITECH Research Group, Universidad El Bosque, Bogota, Colombia; Valle Salud Clinic, Cali, Colombia; Oregon Health & Science University, Portland, Oregon; Oregon Health & Science University, Portland, Oregon; Zucker School of Medicine at Hofstra/Northwell, Hempstead, New York; University of Utah, Salt Lake City, Utah; University of Washington, Seattle, Washington; Stanford University, Stanford, California; University of British Columbia, Vancouver, British Columbia; University of Pittsburgh, Pittsburgh, Pennsylvania; Harvard Medical School & Boston Children's Hospital, Boston, Massachusetts; University of Washington, Seattle, Washington; Drexel University, Pittsburgh, Pennsylvania; Emory University, Atlanta, Georgia; Stanford University, Stanford, California; Stanford University, Stanford, California

**Keywords:** Traumatic brain injury, Head injury, Guideline, Fourth edition, Brain Trauma Foundation, Severe, Decompressive craniectomy, Craniectomy, Decompression, Surgery

## Abstract

When the fourth edition of the Brain Trauma Foundation's Guidelines for the Management of Severe Traumatic Brain Injury were finalized in late 2016, it was known that the results of the RESCUEicp (Trial of Decompressive Craniectomy for Traumatic Intracranial Hypertension) randomized controlled trial of decompressive craniectomy would be public after the guidelines were released. The guideline authors decided to proceed with publication but to update the decompressive craniectomy recommendations later in the spirit of “living guidelines,” whereby topics are updated more frequently, and between new editions, when important new evidence is published. The update to the decompressive craniectomy chapter presented here integrates the findings of the RESCUEicp study as well as the recently published 12-mo outcome data from the DECRA (Decompressive Craniectomy in Patients With Severe Traumatic Brain Injury) trial. Incorporation of these publications into the body of evidence led to the generation of 3 new level-IIA recommendations; a fourth previously presented level-IIA recommendation remains valid and has been restated. To increase the utility of the recommendations, we added a new section entitled *Incorporating the Evidence into Practice.* This summary of expert opinion provides important context and addresses key issues for practitioners, which are intended to help the clinician utilize the available evidence and these recommendations.

The full guideline can be found at: https://braintrauma.org/guidelines/guidelines-for-the-management-of-severe-tbi-4th-ed#/.

**ABBREVIATIONS**
DC: decompressive craniectomy
GOS-E: Glasgow Outcome Scale–Extended
ICP: intracranial pressure
ICU: intensive care unit
OR: odds ratio
RCT: randomized controlled trial
SIBICC: Seattle International severe traumatic Brain Injury Consensus Conference
TBI: traumatic brain injury

Treatment of intracranial pressure (ICP) elevation is central to the management of patients with severe traumatic brain injury (TBI).^1-3^ The volume of the intracranial contents often increases following TBI as a result of hemorrhage, cerebral edema, and hydrocephalus. This can lead to an injurious shift of the brain–termed herniation. Moreover, the increase in volume within the rigid skull can increase ICP leading to a compartment syndrome^4,5^ which impedes or prevents the flow of blood to the brain.^3,6,7^ The ensuing cerebral ischemia can ultimately result in disability or death.

The temporary removal of a large portion of skull–termed decompressive craniectomy (DC)–has long been part of the neurosurgeon's armamentarium for treating ICP elevation resulting from TBI.^8^ Primary DC occurs when the bone flap is not replaced when an intracranial mass lesion is evacuated early after a head trauma. Secondary DC involves the removal of the bone flap later in the patient's course–typically to treat the elevation of ICP refractory to other treatments. Over the last century, the use of DC has been controversial. Technical aspects of the surgery, timing, and patient selection continue to be debated, and there has even been disagreement as to whether this procedure should be performed at all. There had been hope that the RESCUEicp (Trial of Decompressive Craniectomy for Traumatic Intracranial Hypertension) and DECRA (Decompressive Craniectomy in Patients with Severe Traumatic Brain Injury) randomized controlled trials (RCTs) would provide definitive guidance as to if and how this technique should be employed. The results of these studies have defied simple interpretations, however; DC remains controversial despite the publication of these high-quality trials.^9-11^

In the fourth edition of the Brain Trauma Foundation's Guidelines for the Management of Severe Traumatic Brain Injury published in 2017,^12^ the lead chapter provided 2 level-IIA recommendations on the topic of DC. These recommendations served to update the first published clinical practice guidelines for DC provided in conjunction with the Brain Trauma Foundation's Guidelines for the Surgical Management of Traumatic Brain Injury published in 2006.^13^ Here, we present an update of the 2017 recommendations following the adjudication and consideration of the evidence provided by RESCUEicp^11^ as well as DECRA’s recently published 12-mo outcome data.^10^ One of the previous recommendations was retained and 3 new level-IIA recommendations are now provided on this topic.

## UPDATED RECOMMENDATIONS

DECRA^10^ and RESCUEicp^11^ are higher quality studies whose findings supersede those of lesser quality investigations. Though both studied secondary DC for the treatment of refractory ICP elevation, a key difference in the study protocols for DECRA^10^ and RESCUEicp^11^ is that they were designed to investigate conditions of *early* and *late* refractory ICP elevations, respectively. Indeed, DECRA enrolled TBI patients with ICP above 20 mm Hg for 15 min over a 1-h period despite the optimization of tier 1 treatments within the first 72 h of care (early), while RESCUEicp enrolled patients with ICP greater than 25 mm Hg for 1 to 12 h refractory to 2 tiers of treatment within 10 d of admission (late). In constructing the recommendations that follow, we thus refer to *early and late refractory ICP elevations* to reference conditions similar to those studied in DECRA and RESCUEicp, respectively.

The addition of these studies to the available research evidence is the basis for the following updated recommendations:

Level IIA–to improve mortality and overall outcomes

**1.** NEW–Secondary DC performed for *late* refractory ICP elevation is recommended to improve mortality and favorable outcomes.
**2.** NEW–Secondary DC performed for *early* refractory ICP elevation is not recommended to improve mortality and favorable outcomes**†**.
**3.** A large frontotemporoparietal DC (not less than 12 × 15 cm or 15 cm in diameter) is recommended over a small frontotemporoparietal DC for reduced mortality and improved neurological outcomes in patients with severe TBI.

Level IIA–for ICP control

**4.** NEW–Secondary DC, performed as a treatment for either early or late refractory ICP elevation, is suggested to reduce ICP and duration of intensive care, though the relationship between these effects and favorable outcome is uncertain.

**†** *Recommendation #2 should not be extrapolated to primary DC in which the bone flap is left off when an intracranial mass lesion is evacuated early after injury.*

### Changes from Prior Edition

The recommendations for DC in the fourth edition (2017) were as follows:

#### Level IIA

1. “Bifrontal DC is not recommended to improve outcomes as measured by the Glasgow Outcome Scale-Extended (GOS-E) score at 6 months post-injury in severe TBI patients with diffuse injury (without mass lesions), and with ICP elevation to values > 20 mm Hg for more than 15 minutes within a 1-h period that are refractory to first-tier therapies. However, this procedure has been demonstrated to reduce ICP and to minimize days in the intensive care unit (ICU).
2. A large frontotemporoparietal DC (not less than 12 × 15 cm or 15 cm diameter) is recommended over a small frontotemporoparietal DC for reduced mortality and improved neurologic outcomes in patients with severe TBI.”^12^

The first recommendation was based on the 6-mo outcomes from DECRA.^9^ The second recommendation was based on 2 studies: Jiang et al (2005)^14^ and Qiu et al (2009).^15^

By virtue of the updated body of evidence, including 12-mo outcome data from DECRA and RESCUEicp, both published subsequent to the 2017 guidelines, we have removed the first recommendation and restated the second. We also provide 3 new level-IIA recommendations. New recommendation #1 relates to the positive findings of the RESCUEicp study,^11^ while new recommendation #2 relates to the negative findings of the DECRA study.^9,10^ Recommendation #4 reflects findings consistent in both studies.^9-11^

### Evaluation of the Updated Body of Evidence

The scope of this update was limited to the addition of the RESCUEicp study and the 12-mo DECRA outcome data to the existing body of evidence. Following the appraisal and addition of this evidence, we assessed whether changes to the 2017 recommendations were appropriate. The methodology and analytical framework employed were consistent with those used for the previously published fourth edition.^12^

To complete these tasks, we assembled a subcommittee comprising a senior methodologist and 2 clinical investigators who were authors of the DC section of the 2017 guidelines document.^12^ The work of this subgroup was then presented to all authors of the fourth edition guidelines. The updated recommendations were then extensively discussed and revised.

## SUMMARY OF ASSESSMENT TASKS

### Quality of the Body of Evidence

Studies of DC covered several questions (Table 1). The class 1 studies compared DC to medical management, while class 2 studies compared DCs of different sizes in terms of their effect on patient mortality and functional outcomes. Class 3 studies addressed these questions, and also (3) compared DC to craniotomy and (4) assessed the use of DC earlier or later in the course of treatment.^12^

**TABLE 1.** Quality of the Body of Evidence

Topic	Number of studies	Meta-analysis	Number of subjects	Class of studies	Consistency (high, moderate, low)	Directness (direct or indirect)	Precision (high, moderate, low)	Quality of evidence (high, moderate, low, insufficient)
Components of overall quality–classes 1 and 2								
DC vs medical management^9-11^	2 RCTs	NA	553	1	Moderate	Direct	Moderate	Moderate
Larger DC vs smaller DC^12,14^	2 RCTs	No: Different outcomes	560	2	Moderate	Direct	Moderate	Moderate
Components of overall quality–class 3								
DC vs craniotomy^15,16^	2 observational	No	174	3	Moderate	Direct	Low	Insufficient
Timing of DC^17,18^	2 observational	No	160	3	Low	Direct	Low	Insufficient

For the first 2 questions, the overall quality of the body of evidence was moderate. Both RCTs that compared DC to initial medical management were rated class 1.^9-11^ Because of the important differences between these studies, there remains a need to replicate their findings before achieving a high level of confidence that the conclusions will not be changed with the results of future studies. Both RCTs that compared sizes of DCs were rated class 2.^14,15^ The class 3 studies on the questions of DC vs craniotomy and the timing of DC questions were not incorporated into the recommendations and are not included in Table 2, given higher level evidence was available. The class 3 studies remain available for reference in the fourth edition.^12^

**TABLE 2.** Summary of Evidence – Class 1 and 2 Studies of Decompressive Craniectomy

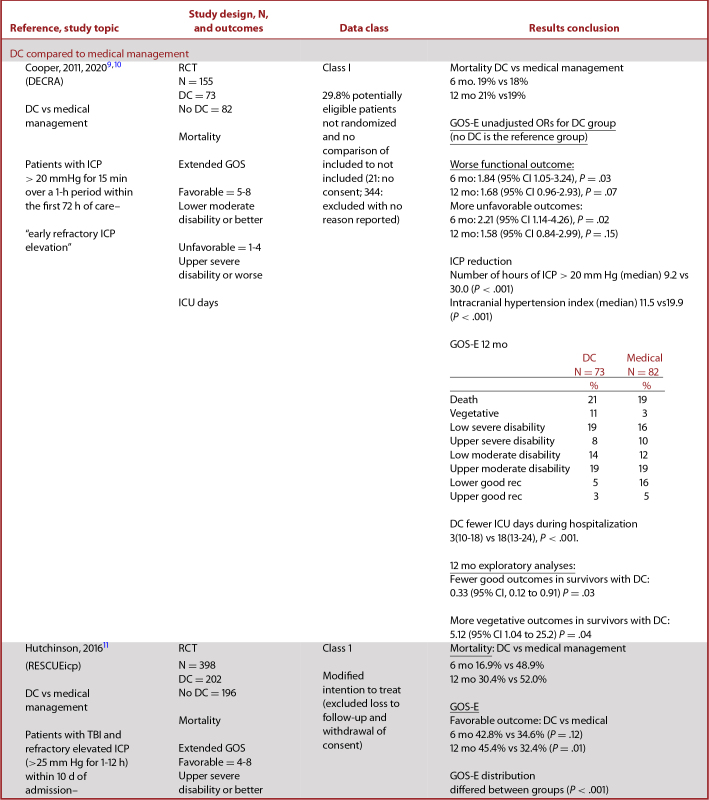
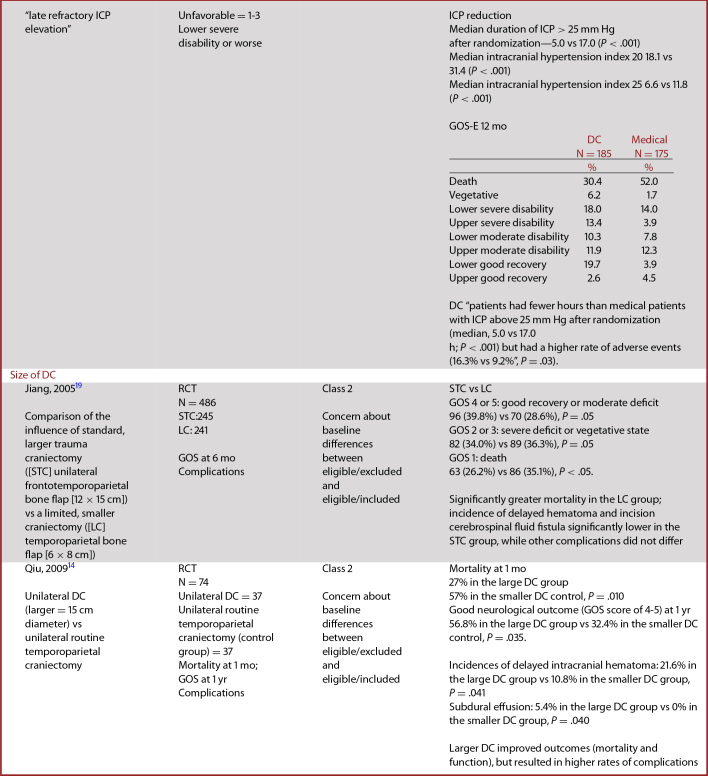

### Quality Assessment

Fundamental to this update was an appraisal of the level of evidence provided by both the DECRA and the RESCUEicp RCTs. DECRA had previously been judged to be a class I study with an overall quality rating of “*Good*” (on a scale of *Good/Fair/Poor*)^12^ as it was an RCT without multiple, serious risks of bias. RESCUEicp underwent an independent assessment for quality by 2 reviewers using the same instrument as used to assess the DECRA trial and all other studies included in the fourth edition. RESCUEicp was rated a good-quality (class I) study as DECRA had been (see **Appendix**, RESCUEicp and DECRA quality assessment). The new DECRA publication^10^ was similarly evaluated, and we determined that the new information provided about the study should not change the rating.^9^

### Data Abstraction

Table 2 contains updated data abstractions for the DECRA and RESCUEicp trials. DECRA and RESCUEicp are both RCTs that compared outcomes from patients receiving DC to outcomes from medical management as interventions for severe TBI with refractory ICP elevation.

### Applicability

Applicability was adjudicated and found to differ across questions and studies. DECRA was conducted in 3 countries over an 8-yr period and included 15 medical centers. RESCUEicp was conducted in 20 countries over a 10-yr period and included 73 medical centers. While the diversity of patients and settings may have limited the ability to detect an effect, it could increase the applicability of the studies.

The 2 class 2 studies that compare sizes of DCs were both conducted in one country (China).^14,15^ These publications provide incomplete information on key details, limiting our ability to adjudicate elements such as the standard of care and characteristics of the studied populations.

## SUMMARY OF THE EVIDENCE

### Class 1 and 2 Studies

The characteristics and results of class 1 and 2 studies of DC are summarized in Table 2.

### Evidence Summary

The relationship between secondary DC and neurological outcome following severe TBI has been investigated in many studies; however, 2 RCTs provide the best evidence currently available. The 2 studies are DECRA^9,10^ and RESCUEicp,^11^ both providing class I evidence.

#### DECRA

DECRA compared outcomes for patients with diffuse brain injury treated with early bifrontal DC to those treated with medical management. Patients with intracranial mass lesions were excluded from enrollment. They used 3 primary methods for comparison. (1) They compared group differences in control of ICP, days of mechanical ventilation, days in intensive care unit (ICU), and mortality. (2) They used the median score for each group on the 8-item GOS-E scale to calculate the odds of worse outcomes. (3) They dichotomized the 8-item GOS-E scale to calculate the odds of unfavorable outcomes.

Using group differences assessed at 6 mo postenrollment–the primary outcome–they found the DC group had lower ICP, fewer days on mechanical ventilation and in the ICU, and no difference between groups for mortality. Using the median score for each group of the GOS-E measured at 6 mo postinjury (3: DC, 4: No DC), the unadjusted odds ratio (OR) for worse outcomes in the DC group was 1.84 (95% CI 1.05-3.24), *P* = .03, but after adjustment, the OR was no longer significant. Using the dichotomized score (1-4 vs 5-8), both unadjusted and adjusted odds of unfavorable outcomes were significantly greater in the DC group.

More recently, the DECRA investigators published the 12-mo outcome data from their study. This has been highly anticipated as there is a growing sense that assessment at 6 mo may be a premature endpoint for the severe TBI population; moreover, it facilitates a comparison to the 12-mo outcome data provided by RESCUEicp. Notably 12-mo outcome data were a secondary endpoint in both trials, however.

The 12-mo outcome data from DECRA were similar to what was noted at 6 mo. At 12 mo, there was a trend to worse functional outcomes in the craniectomy group (OR 1.68, 95% CI 0.96-2.93; *P* = .07) as well as unfavorable functional outcomes (OR 1.58; 95% CI 0.84-2.99; *P* = .16), though these results were not statistically significant as they were in the 6-mo data. An exploratory post-hoc analysis noted that amongst survivors after craniectomy, there were fewer good (OR 0.33; 95% CI 0.12- 0.91; *P* = .03) and more vegetative (OR 5.12; 95% CI 1.04-25.2; *P* = .04) outcomes.

#### RESCUEicp

RESCUEicp compared outcomes of patients who received DC as a salvage treatment for ICP elevation with those who received medical management. It tolerated ICP elevation for a longer duration prior to enrollment than the DECRA study. RESCUEicp was intended to study patients with intracranial mass lesions and those undergoing lateral decompressions. However, the study predominantly enrolled patients with diffuse injuries, and most patients underwent bifrontal decompressions, making the included patients very similar to those enrolled in DECRA.

RESCUEicp used 2 methods for comparison. (1) They compared group differences in control of ICP, mortality, and distribution of the GOS-E ratings. (2) They dichotomized the 8-item GOS-E scale to compare favorable vs unfavorable outcomes between groups. Using group differences, for the primary outcome measured at 6 mo postinjury, there was a lower rate of mortality and higher rate of vegetative state, lower severe disability, and upper severe disability in patients in the DC group. The unordered test comparing the distribution of the GOS-E ratings over the 2 groups yielded a χ^2^ of 30.69 (7 df, *P* < .001) (individual *P* values not reported). Using the dichotomized score (1-3 vs 4-8, a different dichotomization from that used in DECRA), 42.8% of patients in the DC group had favorable outcomes vs 34.6% of patients in the No DC group (*P* = .12). At 12 mo, this same comparison of favorable outcomes reached statistical significance with 45.4% of patients in the DC group with favorable outcomes vs 32.4% of patients in the No DC group (*P* = .01).

As with DECRA, RESCUEicp demonstrated that DC effectively lowered ICP and reduced the duration of intensive care management. It demonstrated an increase in the rate of poor outcomes when comparing the distribution of GOS-E ratings, but no difference in outcomes using the dichotomized GOS-E. Of note, for the secondary outcome at 12 mo postinjury, significantly more patients in the DC group than the No DC group had favorable outcomes, using the dichotomized score of GOS-E (*P* = .01).

#### Comparability of DECRA and RESCUEicp

Comparability of the outcomes from these 2 studies is compromised by a number of factors. The design of DECRA targeted the effects of DC as applied to early stages of resistant intracranial hypertension, whereas RESCUEicp studied patients with more established resistance. Time from injury to treatment was lower in DECRA than in RESCUEicp. The ICP treatment threshold was higher and duration of time above that threshold was longer in RESCUEicp than in DECRA. The surgical approach varied across the 2 studies and within RESCUEicp. Finally, they used different cut-points for the dichotomization of GOS-E, increasing the probability of a higher proportion of patients with favorable outcomes in RESCUEicp.

DECRA and RESCUEicp were consistent in demonstrating that DC reduces ICP and duration of intensive care. They both also detected an increased rate of some levels of poor outcomes with DC. Although it used a secondary outcome measure with a more generous dichotomization scheme, 12-mo data in RESCUEicp seem to indicate that the outcome benefits of decompression continue to improve beyond the pre-specified six-month test period. This same effect is suggested in the DECRA study as the ORs had smaller magnitudes and *P*-values became nonsignificant at 12 mo; however, this is best interpreted as a reduction in the magnitude of the harm associated with DC. Findings related to mortality were inconsistent between the studies with a mortality benefit noted in RESCUEicp but none in DECRA.

The authors debated the extent to which the bifrontal surgical procedures performed in the DECRA and RESCUEicp studies should be extrapolated to the lateral decompressions more popular in North America. In DECRA, bifrontal decompressions were performed exclusively, and they were performed more commonly than lateral decompressions in RESCUEicp. Given a lack of evidence discriminating these 2 forms of DC at this time, we chose to provide recommendations that do not reference a specific decompressive surgery. It will be desirable in the future, however, to determine if there are differences in the risk:benefit ratios of these surgeries and whether one or the other should be applied preferentially in specific circumstances.

## EXPERT OPINION

### Incorporating the Evidence Into Practice

#### Translation of Evidence Into Practice

DC remains a very controversial topic in the TBI field. There was much hope that the 2 key RCTs, DECRA and RESCUEicp, would provide clarity on whether or not this is a procedure that neurosurgeons should perform amongst other therapeutic options available for severe TBI. Unfortunately, neither DECRA nor RESCUEicp, together or separately, provides definitive evidence for or against the performance of DC, and they are both complex and challenging to interpret. Perhaps the most important conclusion of these studies is that choosing to perform a DC is not a simple decision and that the potential benefits should be balanced against the complications and likely outcomes on a case-by-case basis.

#### Impact of Evidence on Practice

Anecdotal evidence suggests that these new RCTs have not markedly changed practice. DC continues to be performed. This likely reflects uncertainty in the prognosis of individual patients as well as the fact that it is hard to withhold a possibly life-saving therapy even when the odds of functional recovery are believed to be low. Indeed, despite important advances in our ability to predict prognosis on a population level for severe TBI patients early after injury,^16,17^ it remains hard to accurately predict outcomes for an individual patient.

#### Recent Clinical Consensus

Subsequent to the publication of RESCUEicp, a large international consensus conference was convened in an effort to provide recommendations for the use of DC in severe TBI care. Expert opinion and experience were used to address the gaps in evidence. The proceedings of this conference were recently published.^18^ The consensus conference concluded that DC remains a treatment option for severe TBI patients and that both bifrontal and lateral decompressions are reasonable to perform. However, the group also agreed that DC should not be applied indiscriminately and that additional efforts to assess the likelihood of positive patient outcomes should be made prior to DC. For example, preoperative magnetic resonance imaging scans could reveal devastating structural brain lesions (such as in the brainstem) not seen on computed tomography, which would predict a lack of benefit from surgical decompression. Patients with evidence of “good” brain function who decline as a direct consequence of ICP elevation are likely the best candidates for decompression; however, identifying such patients remains challenging. The group also recognized that longer term outcome measures are important in future studies commensurate with the ongoing clinical improvements in severe TBI patients from 6 to 24 mo postinjury. These recommendations are reflected in the inclusion of DC as a tier 3 treatment option in the Seattle International severe traumatic Brain Injury Consensus Conference (SIBICC) management algorithms.^1,2^

#### What Is a “Good” Outcome?

Central to the debate around the performance of DC is the issue of what constitutes acceptable (or “good”) neurological recovery. Of additional complexity is the question of what is acceptable precision in predicting this “acceptable” recovery. Different cultures, families, and patients define what is meaningful function and what makes life worth living differently; the answers to these questions are more philosophical than medical. Given the controversy surrounding DC and the uncertainty about which patients will return to prior or meaningful function and which will not, family members or other proxy decision-makers familiar with patients’ values and preferences should be provided with the best information available and included in clinical decision making.

### Future Directions

Though convincing evidence currently supports that DC reduces ICP, and that DC of insufficient size is associated with poor outcomes, additional high-quality studies are needed to inform every aspect of DC as it is applied in clinical practice for severe TBI. Several particularly important gaps in knowledge are evident from this analysis of the body of evidence. The relative risks and benefits of lateral DC as compared to bifrontal DC are a critical knowledge gap. It remains to be seen if the decompression should be tailored to the intracranial pathology. For instance, one might hypothesize that a bifrontal DC is better suited to a patient with frontal contusions, while a lateral decompression may be preferable for those with extra-axial hematomas but this remains uninformed by evidence. The importance of incising the falx when a bifrontal DC is performed was questioned in conjunction with the interpretation of the DECRA trial, but this too remains insufficiently understood. It has been suggested that efforts should be made to better identify patients who will benefit from DC and that additional investigations may assist this process.^18^ This should be a very high priority for ongoing and new research efforts. There is also a growing sense that the traditional trial endpoint–the GOSE score at 6 mo–assesses outcomes prematurely and that longer term follow-up would be preferred in severe TBI treatment studies. The optimal time to perform bone flap replacement–or cranioplasty–is also insufficiently understood at present.

It is also important to consider that current literature predominantly relates to secondary DC, in which the bone flap is removed in a delayed fashion to treat refractory elevation of ICP. A paucity of literature currently informs primary DC, or the practice of leaving the bone flap off following an initial surgery to evacuate an intracranial mass lesion. It is hoped that the ongoing RESCUE-SDH RCT will help inform the performance of primary DC with high-quality data.^19,20^

### Disclosures

This material is based upon work supported by (1) the US Army Contracting Command, Aberdeen ProvingGround, Natick ContractingDivision, through a contract awarded to Stanford University (W911 QY-14-C-0086). Any opinions, findings, and conclusions, or recommendations expressed in this material are those of the authors and do not necessarily reflect the views of the US Army Contracting Command, Aberdeen Proving Ground, Natick Contracting Division, or Stanford University. The authors have no personal, financial, or institutional interest in any of the drugs, materials, or devices described in this article. This material is based upon work supported by (1) the US Army Contracting Command, Aberdeen ProvingGround, Natick ContractingDivision, through a contract awarded to Stanford University (W911 QY-14-C-0086). Any opinions, findings, and conclusions, or recommendations expressed in this material are those of the authors and do not necessarily reflect the views of the US Army Contracting Command, Aberdeen Proving Ground, Natick Contracting Division, or Stanford University. The authors have no personal, financial, or institutional interest in any of the drugs, materials, or devices described in this article.

## Supplementary Material

nyaa278_Supplemental_Appendix

## Notes

Reviewed for evidence-based integrity and endorsed by the American Association of Neurological Surgeons and Congress of Neurological Surgeons.

## Notes

**Appendix.** RESCUEicp and DECRA Quality Assessment.
