# Supplementary material for: Guidelines for the Management of Severe Traumatic Brain Injury: 2020 Update of the Decompressive Craniectomy Recommendations
Source: Neurosurgery. 2020 Aug 6;87(3):427–34. doi: 10.1093/neuros/nyaa278 (PMC7426189; doi:10.1093/neuros/nyaa278)
Supplement: nyaa278_Supplemental_Appendix [file nyaa278_supplemental_appendix.docx]

| **Author, Year** | **Was randomization adequate?** | **Was the allocation of treatment adequately concealed?** | **Outcome assessors blinded?** | **Care provider blinded?** | **Patient blinded?** | **Were groups similar at baseline?** | **Was loss to  follow up 20%  or less?** | **Was loss to follow up similar across groups? (that is were comparable groups maintained)** | **Was intention-to-treat analysis used?** | **Were outcomes pre specified and were primary/pre specified outcomes reported?** | **Were outcomes assessed using valid and reliable measures?** | **Was the study registered?** | **Quality Rating** |
| --- | --- | --- | --- | --- | --- | --- | --- | --- | --- | --- | --- | --- | --- |
|  | *Yes/No/Method NR* | *Yes/No/ Unclear/NR* | *Yes/No/ Unclear* | *Yes/No/ Unclear* | *Yes/No/ Unclear* | *Yes/No/Unclear* | *Yes/No* | *Yes/No/Unclear* | *Yes/No/Unclear* | *Yes/No/Unclear* | *Yes/No/Unclear* | *Yes/No* | *Good/Fair/Poor* |
| Cooper 2011, 2020 ^9,10^ | Yes | Yes | Yes | No | No | There were significantly fewer patients with reactive pupils in DC group (p = 0.04). However, a post hoc adjustment was performed, which resulted in no significant difference in GOS-E Scores between groups. | Yes | Yes | Yes | Yes | Yes | Yes | Good |
| Hutchinson 2016  ^11^ | Yes | Yes | Yes | No | No | More drug/alcohol use in No DC group. Significant difference not reported, and no adjustments were made. | Yes | Yes | Modified intention to treat (excluded loss to follow-up or withdrawal of consent). However, excluded patients constituted < 10% per group, and not considered a serious threat to internal validity. Loss at 6 mos: DC N = 5 No DC N = 12 | Yes | Yes | Yes | Good |

**Appendix.** RESCUEicp and DECRA Quality Assessment.
